# Comparing clinical decision-making of AI technology to a multi-professional care team in an electronic cognitive behavioural therapy program for depression: protocol

**DOI:** 10.3389/fpsyt.2023.1220607

**Published:** 2023-12-21

**Authors:** Callum Stephenson, Jasleen Jagayat, Anchan Kumar, Paniz Khamooshi, Jazmin Eadie, Amrita Pannu, Dekel Meartsi, Eileen Danaee, Gilmar Gutierrez, Ferwa Khan, Tessa Gizzarelli, Charmy Patel, Elnaz Moghimi, Megan Yang, Amirhossein Shirazi, Mohsen Omrani, Archana Patel, Nazanin Alavi

**Affiliations:** ^1^Department of Psychiatry, Faculty of Health Sciences, Queen’s University, Kingston, ON, Canada; ^2^Centre for Neuroscience Studies, Faculty of Health Sciences, Queen’s University, Kingston, ON, Canada; ^3^Department of Psychology, Faculty of Arts and Sciences, Queen’s University, Kingston, ON, Canada; ^4^OPTT Inc., Toronto, ON, Canada

**Keywords:** mental health, depression, psychotherapy, eHealth, cognitive behavioural therapy, artificial intelligence, treatment, major depressive disorder

## Abstract

**Introduction:**

Depression is a leading cause of disability worldwide, affecting up to 300 million people globally. Despite its high prevalence and debilitating effects, only one-third of patients newly diagnosed with depression initiate treatment. Electronic cognitive behavioural therapy (e-CBT) is an effective treatment for depression and is a feasible solution to make mental health care more accessible. Due to its online format, e-CBT can be combined with variable therapist engagement to address different care needs. Typically, a multi-professional care team determines which combination therapy most benefits the patient. However, this process can add to the costs of these programs. Artificial intelligence (AI) has been proposed to offset these costs.

**Methods:**

This study is a double-blinded randomized controlled trial recruiting individuals experiencing depression. The degree of care intensity a participant will receive will be randomly decided by either: (1) a machine learning algorithm, or (2) an assessment made by a group of healthcare professionals. Subsequently, participants will receive depression-specific e-CBT treatment through the secure online platform. There will be three available intensities of therapist interaction: (1) e-CBT; (2) e-CBT with a 15–20-min phone/video call; and (3) e-CBT with pharmacotherapy. This approach aims to accurately allocate care tailored to each patient’s needs, allowing for more efficient use of resources.

**Discussion:**

Artificial intelligence and providing patients with varying intensities of care can increase the efficiency of mental health care services. This study aims to determine a cost-effective method to decrease depressive symptoms and increase treatment adherence to online psychotherapy by allocating the correct intensity of therapist care for individuals diagnosed with depression. This will be done by comparing a decision-making machine learning algorithm to a multi-professional care team. This approach aims to accurately allocate care tailored to each patient’s needs, allowing for more efficient use of resources with the convergence of technologies and healthcare.

**Ethics:**

The study received ethics approval and began participant recruitment in December 2022. Participant recruitment has been conducted through targeted advertisements and physician referrals. Complete data collection and analysis are expected to conclude by August 2024.

**Clinical trial registration:**

ClinicalTrials.Gov, identifier NCT04747873.

## 1 Introduction

Depression is a leading cause of disability, affecting approximately 3.8% of the population worldwide (1, 2). Despite the high prevalence and negative consequences of depression, only one-third of individuals receive treatment, and three in five receive sufficient care (3, 4). In 2018, mental health care needs for 2.3 million Canadians were reported to be insufficient with 78.2% indicating reasons relating to accessibility (e.g., lack of knowledge on how to get help, financial issues, and time restraints) to be the top barrier to receiving mental health care (5). Another major hindering factor in patients’ access to effective mental health support is the cost of care (6). This outlines the importance of developing evidence-based and cost-effective solutions to address these issues on mental health care accessibility and efficiency.

Electronic cognitive behavioural therapy (e-CBT) is a good candidate to make mental health care more accessible and is effective in treating various mental health issues including mood and anxiety disorders (7–11). However, this internet-based treatment presents problems of high dropout rates and non-adherence (12). Treatment compliance is an important factor for positive psychotherapy treatment outcomes (13, 14). Drop-out rates for in-person psychotherapy vary from 20–47% depending on the study design (15–17), whereas a systematic review of 29 studies revealed the dropout rate for e-CBT for depression can range from 0–63%, with an average dropout rate of 32% (18). Multiple factors can affect patient compliance such as patient age, education, treatment engagement, and clinician involvement (12, 19, 20).

Furthermore, while pre-designed e-CBT content can make mental health care more scalable and affordable and address the general concerns of most individuals with mild to moderate symptoms (8, 9, 21, 22), individuals with severe symptoms may require further intensive clinical interventions and clinician involvement (22, 23). Stratified care begins treatment by matching care intensity (i.e., low vs. high intensity) based on the patient’s needs. Stratified psychiatry is beneficial because it can increase treatment response and remission rates by allocating treatments effectively (24). An important challenge in using a stratified care strategy is recognizing which group each patient belongs to and which resources are required to address their needs.

Currently, clinical decision-making is conducted by a multi-professional care team which can consist of a psychiatrist, residents, nurses, clinical psychologist, social worker, counsellor, and support worker (25). Although this collaboration is beneficial in many respects (26), is typically costly and results in long wait times (6). A previous framework using real patient data has demonstrated the cost-effectiveness of using machine learning in clinical decision-making (27). The use of machine learning in healthcare has significantly increased over the past few years. In the context of clinical decision-making, machine learning algorithms use large data sets from an array of sources to assist healthcare providers in making rapid and informed decisions (28). To further understand symptom severity and increase treatment efficacy, many researchers have tried to implement supervised machine-learning approaches to identify patient characteristics that may be associated with poor treatment outcomes and develop a treatment for patients (29–32). These novel decision-making algorithms aim to mimic human decision-making to help with objective and accurate evaluation of each patient’s needs (33). This technology can make personalized care a possibility, particularly in cases where clinical decision-making by a care team is not feasible or results in prolonged wait times. Overall, the novelty of machine learning in healthcare demands greater exploration and understanding of responsible use (34).

The goal of this study is to develop a machine learning algorithm that can identify the intensity of care an individual needs based on their probability of dropping out from treatment. This algorithm is expected to be comparable to the clinical decision-making process. It is expected to indicate the appropriate level of care for an individual experiencing depression to make treatment efficient and effective. We believe that the integration of AI-driven clinical decision-making would make mental health care delivery more scalable, accessible, and affordable while remaining highly personalized and effective. To make this possible, we need to (1) make objective and quantified evaluations of patients’ mental status and their needs, (2) utilize a decision-making algorithm that allocates the right level of care for each patient, and (3) demonstrate that this method of care delivery can enhance the quality of mental health care and reduce costs.

## 2 Objectives

The primary objective of this study is to evaluate the effectiveness of the suggested level of care made by the AI compared to the healthcare team and the effectiveness of the stratified care model. This will be assessed using the change in the severity of depressive symptoms using the Patient Health Questionnaire – 9 Item (PHQ-9) (35) and Quick Inventory of Depressive Symptoms (QIDS) (36) scores, and the quality of life and functioning using Assessment of Quality of Life (AQoL-8D) (37) scores between the start and the end of the 13-session e-CBT program.

The secondary objectives include evaluating compliance and treatment completion using the number of sessions completed by each participant and evaluating the time and cost commitment of initial assessments (i.e., manual participant stratification by the healthcare team vs. the AI) and the different care intensities (i.e., only e-CBT, e-CBT + phone calls, or e-CBT + video calls). We will also evaluate the functional consequence of our interventions on participant quality of life via Quality-adjusted life years (QALY)s (38). Throughout the study, QALY estimates will be derived from AQoL-8D scores using the area under the curve method (39, 40). Outlined below are the research questions and the corresponding study hypotheses:

1 Will participants with major depressive disorder (MDD) assigned to an e-CBT program by an AI show similar outcomes in depressive symptoms at 3 months to those allocated to an e-CBT program decided by a team of healthcare professionals?

– We hypothesize that the outcomes in depressive symptoms of treatment arm 1 will be comparable to the outcomes of treatment arms 2 and 3 following e-CBT treatment.

2 How does AI-based clinical decision-making compare to that of a multi-professional healthcare team when allocating individuals with depression to an e-CBT program with different degrees of care intensity?

– We hypothesize that AI-based clinical decision-making (treatment arm 2) will provide suggestions comparable to that of a multi-professional care team (treatment arm 1).

3 Is this novel AI approach to e-CBT time and cost-efficient and comparable to the standard multi-professional healthcare decision-making team?

– We hypothesize that the AI approach will decrease the overall time and cost commitment of providing e-CBT.

## 3 Methods and analysis

### 3.1 Participants

Participants (*n* = 186: *n* = 31 per e-CBT group * 2 arms) will be recruited at Queen’s University from outpatient psychiatry clinics at both Kingston Health Sciences Centre sites (Hotel Dieu Hospital and Kingston General Hospital), as well as Providence Care Hospital in Kingston, Ontario. Additionally, self-referrals and referrals from family doctors, physicians, and clinicians across Ontario will be accepted. After obtaining informed consent from the participant, the participant will be evaluated using the Mini International Psychiatric Assessment (MINI) through a secure video appointment to confirm a diagnosis of MDD using the Diagnostic and Statistical Manual of Mental Disorders, Fifth Edition (DSM-5) (41), by a trained professional on the research team.

All eligible participants will be randomized to receive a treatment plan based on the decision of either the healthcare team (Arm 1) or the Triage Module using an AI algorithm (Arm 2). Participants will be randomly allocated to one of the two arms of the study by a research assistant on the team who will also balance the group based on demographic variables (i.e., sex, gender, age, and income). Participants and therapists in the study will be blinded to which treatment arm the participant belongs to. By the nature of this study, participants and therapists will not be blinded to which treatment intensity the participant will receive since it will be evident whether the participant is receiving a phone/video call in addition to usual e-CBT care or pharmacotherapy. Each participant will be provided with an effective form of treatment (i.e., e-CBT) regardless of which group they will be allocated to. Participants will be informed that there is no incentive to join the program and that joining or withdrawing at any point will not affect them negatively. It will also be explained to the participants that the program is not a crisis resource and that they will not always have access to their therapists. In the case of an emergency, participants will be directed to proper resources, and this event will be reported to the study’s lead psychiatrist (principal investigator). All data will be anonymized and analyzed by research team members who are not directly involved in the patient’s care.

### 3.2 Inclusion and exclusion criteria

The inclusion criteria for this study are at least 18 years of age, diagnosed with MDD according to the Mini International Neuropsychiatric Interview 7.0.2 (MINI) that will be conducted by a trained research assistant. The MINI is a diagnostic interview that assesses 17 common mental disorders by following the diagnostic criteria of the Diagnostic and Statistical Manual of Mental Disorders, 5th edition (DSM-5) (41), ability to provide informed consent, ability to speak and read English, and having consistent and reliable access to the internet. The exclusion criteria include active psychosis, acute mania, severe alcohol, or substance use disorder, and active suicidal or homicidal ideation. Individuals with these disorders are less prone to having good insight into their thoughts; thus, e-CBT on their own may not be fit for them and would be better suited with therapist support throughout therapy. As for individuals with active suicidal or homicidal ideation, this program is not a crisis resource and individuals will be redirected to immediate support and resources that will better assist them. If a participant is receiving or has received CBT or e-CBT in the past year, they will also be excluded from the study to avoid confounding effects on the efficacy of this e-CBT program. If interested, participants excluded from the study will be linked to the appropriate resources.

### 3.3 Study design

**Figure 1:**
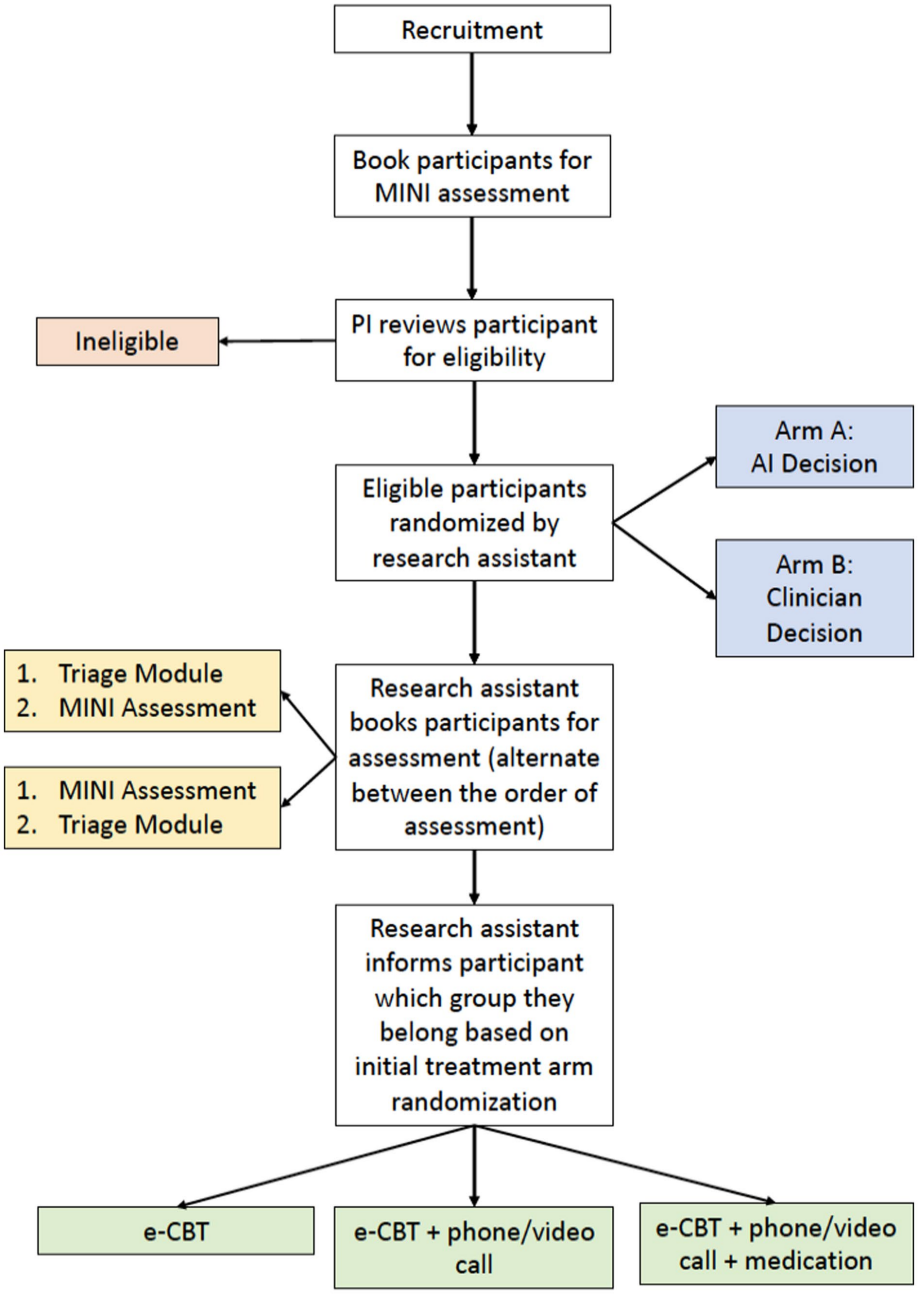
Study design flowchart for enrolment, arm allocation, and treatment intensity decision.

If eligible for this randomized controlled trial, participants (*n* = 186) will be randomized (Figure 1) to receive an e-CBT treatment recommended by a multi-professional healthcare team consisting of a psychiatrist, psychiatric medical resident, and a trained research assistant (Arm 1, control group; *n* = 93), or the AI machine learning algorithm (Arm 2, experimental group; *n* = 93; Figure 1). To ensure blinding, all participants will complete the intake assessment by the healthcare team (Arm 1) and the Triage Module (Arm 2). Only the relevant data (i.e., Arm 1: intake assessment vs. Arm 2: Triage Module) will be analyzed depending on the treatment arm that the participant is randomly assigned to.

#### 3.3.1 Treatment arm 1: healthcare team allocation

Allocation of treatment intensity by the multi-professional healthcare team will be based on the following criteria:

1. The severity of symptoms/disability (DSM-5 criteria).
2. Mental health factors (prior treatments and responses, current and past psychotic/manic episodes, current and past suicidal/homicidal ideation/attempts, family mental health history, past psychiatric history, and hospital admissions).
3. Medical factors (current medical conditions and medications, personal and family medical history).
4. Social factors (support system and living situation, and occupational, social, and personal functional impairment).

To assess the severity of MDD symptoms and the functional impairments, participants will complete the PHQ-9 and Sheehan Disability Scale (SDS) (42, 43) before the assessment appointment. The trained research assistant on the multidisciplinary team will conduct the assessment appointment and will relay the information to the rest of the team later to deliberate on treatment intensity allocation. All assessments will occur virtually through phone and video calls. Together, the healthcare team will decide whether the participant should be assigned to the e-CBT-only treatment, e-CBT treatment with weekly phone/video calls, or e-CBT treatment with pharmacotherapy. This process mimics the current triage process in clinical settings. To track cost-effectiveness, the trained research assistant will track the total duration of the individual assessment and team deliberation meetings for analysis of the total time commitment per patient.

#### 3.3.2 Treatment arm 2: AI algorithm allocation

Allocation of treatment intensity by the proposed AI algorithm will be based on the machine learning and natural language processing (NLP) of textual data provided by participants and their PHQ-9 score collected through a pre-treatment screening module called the Triage Module. This module, developed by the research team, (1) provides psychoeducation on the effects of psychotherapy, (2) collects PHQ-9 scores, and (3) asks participants six open-ended questions regarding their mental health history, their experiences with mental health disorders, and what mental health difficulties they are currently facing. Based on the participant’s answers to the open-ended questions, a variable called “Symptomatic Score” will be calculated using the NLP algorithm. If the PHQ-9 score < 19 and the Symptomatic Score > 0.75, the participant will be assigned to the e-CBT-only treatment group. However, if either the PHQ-9 score is >19 or the Symptomatic Score is <0.75, the participants will be assigned to the e-CBT treatment with weekly phone/video calls. If both scenarios occur and the PHQ-9 > 19 and Symptomatic Score < 0.75, then the participant will be assigned to the e-CBT treatment with pharmacotherapy. This NLP algorithm is patented by OPTT Inc. (International Patent System, PCT/US22/43514).

To gather the relevant data (i.e., participant compliance and change in depression severity, as evaluated by the PHQ-9), the Triage Module was designed. As previously explained, NLP of the participants’ written accounts of their challenges with depression in the Triage Module will be used to calculate a Symptomatic Score. To verify the AI’s treatment allocation logic, completion rate and the change in PHQ-9 scores in a sample of participants (*n* = 190) who were previously enrolled in e-CBT-only treatment was assessed. The decision-making algorithm determined that the e-CBT-only program was suitable for 62 of the 190 participants (32.63%). Within these 62 participants, 53.22% (*n* = 33) had completed the e-CBT-only program in its entirety and only 20% (*n* = 12) had a final PHQ-9 score > 14. Furthermore, the algorithm indicated that e-CBT with telephone calls would be suitable for 100 out of the 190 participants (52.63%). Of the 100 participants, 41.0% (*n* = 41) completed the whole round of e-CBT-only therapy and 31.0% (*n* = 31) had a final PHQ-9 score > 14. Lastly, the algorithm indicated that e-CBT with video call was appropriate for 28 out of 190 participants (14.74%). Of these 28 participants, 35% (*n* = 10) completed the whole round of e-CBT-only therapy and 40% (*n* = 11) had a final PHQ-9 score > 14. The logic of the AI’s decision is therefore justified as those participants allocated to the e-CBT-only group had the highest percentage of completion and lowest percentage of final PHQ-9 scores >14 when completing e-CBT-only. Therefore, minimal therapist intensity is required for these individuals and e-CBT-only is sufficient. Conversely, participants allocated to the e-CBT with video call had the lowest completion rates and highest rates of final PHQ-9 scores >14 when enrolled in e-CBT-only. These findings justify the AI’s logic that greater therapist interaction is required. It is also important to note that demographic factors like age (below or above 40 years), biological sex (male or female) and income (less or more than CAD 50,000) did not have any significant effects on the number of sessions completed by participants (*p* = 0.92, 0.18, 0.90 for age, sex, and income respectively). The demographic factors did not affect the change in PHQ-9 score (i.e., the difference between the beginning and end of treatment scores) either (*p* = 0.20, 0.46, 0.39 for age, sex, and income respectively).

### 3.4 Treatment intensities

**Figure 2:**
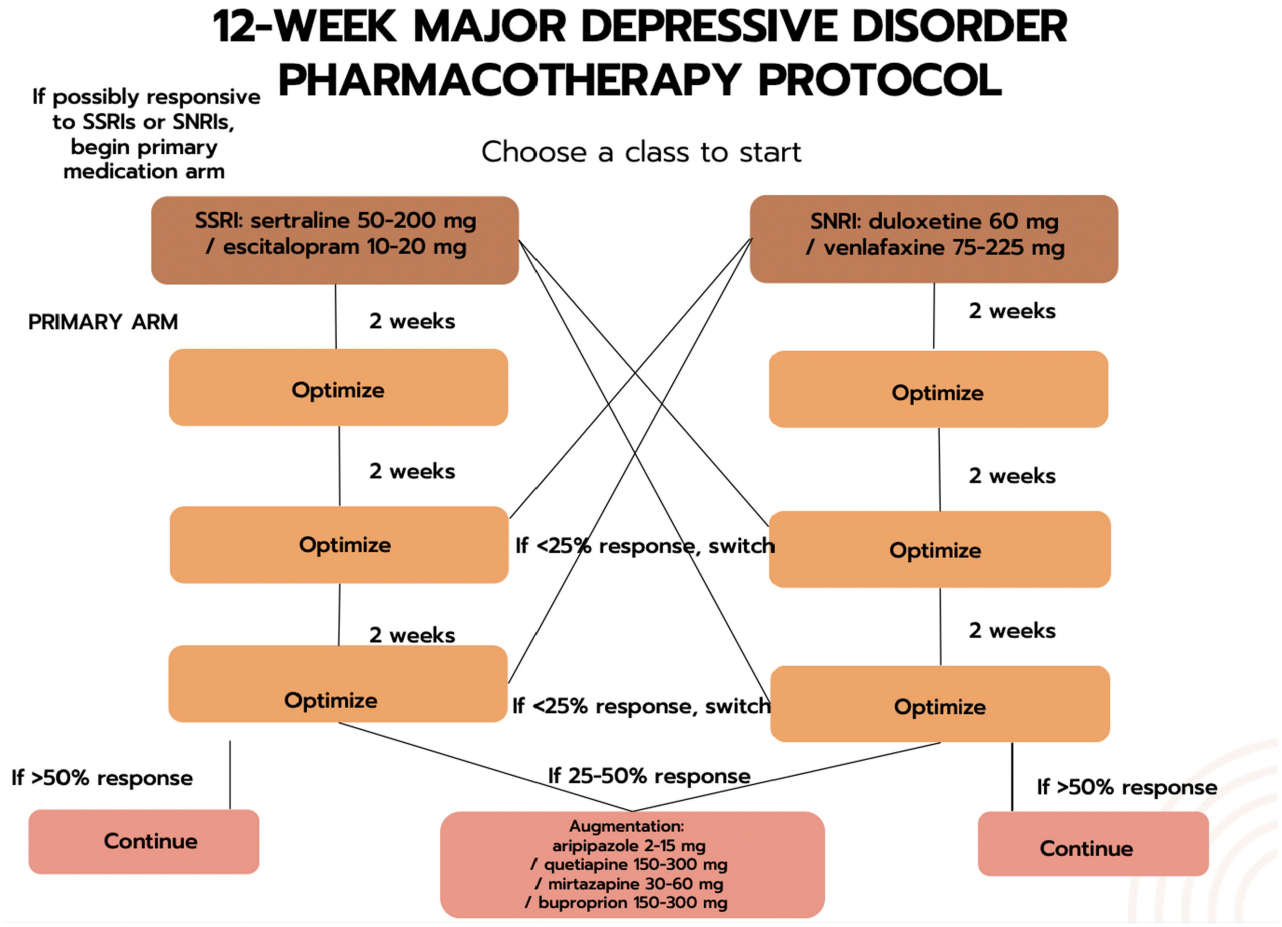
The first phase of the drug-allocation process.

**Figure 3:**
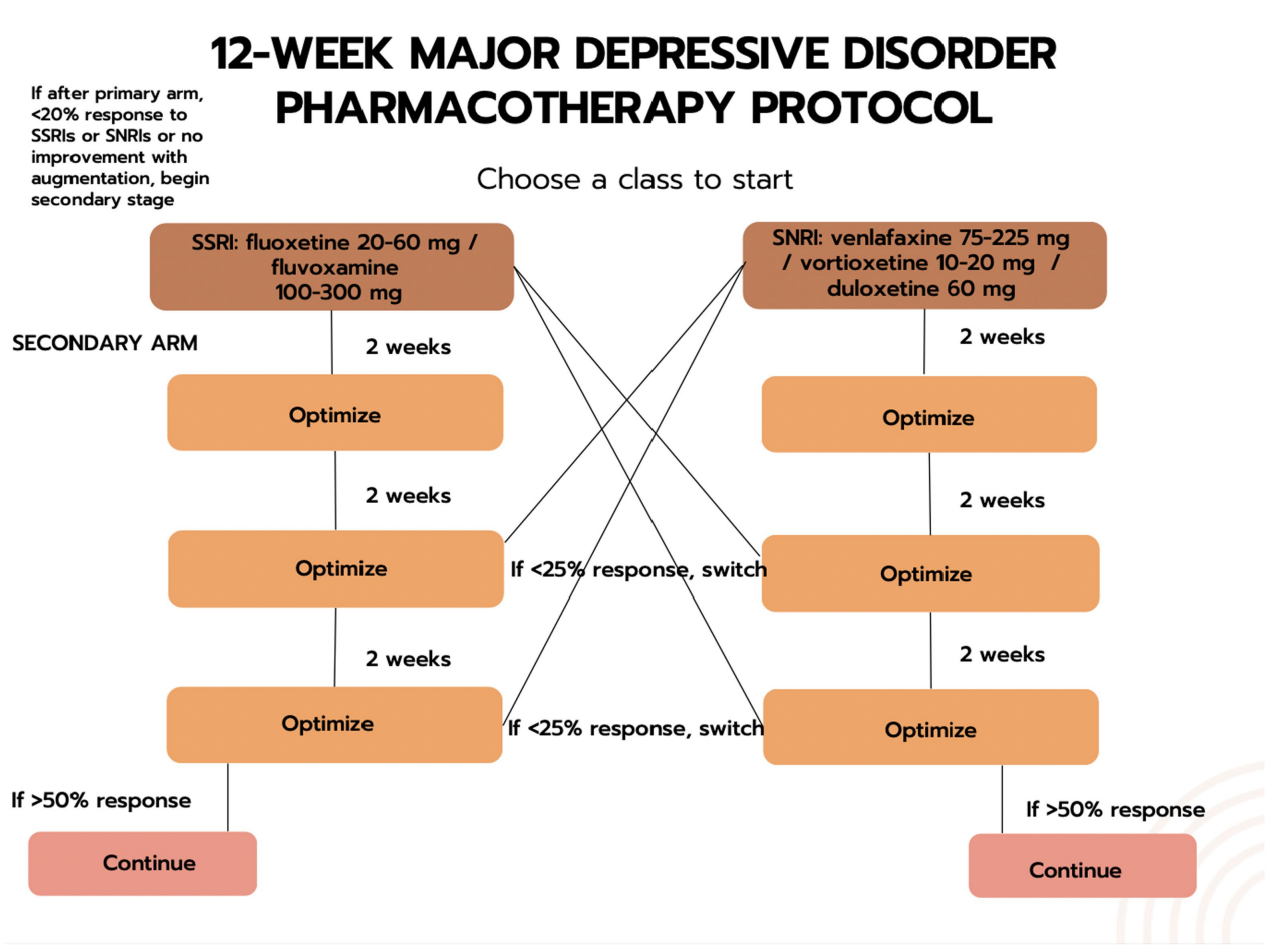
The second phase of the drug-allocation process.

The three treatment intensities decided by the treatment arms (i.e., Arm 1: intake assessment or Arm 2: triage module using an AI algorithm) are:

1. e-CBT Program: The participant will submit their weekly homework and receive personalized feedback from their assigned therapist on the Online Psychotherapy Tool (OPTT). The feedback adds customization by acknowledging the participant’s experiences in the past week and ensures the participant has understood the CBT concepts.
2. e-CBT Program + Call: In addition to the e-CBT program (see 1 above), the participant will receive a weekly phone/video call from their assigned therapist. The goal is to build on the therapeutic relationship and to add personalization with direct verbal encouragement. This phone call is limited to a one-time, 15–20-min call each intervention week (44). The purpose is to check with the patient on their treatment progress. The call will either be a secure phone or a video call, depending on the preference of the patient.
3. e-CBT Program + Pharmacotherapy: In addition to the e-CBT program (see 1 above), the participant will receive standard pharmacotherapy following DSM-5 guidelines. A pharmacotherapy allocation system has been developed (Figures 2, 3) that follows clinical guidelines.

While pharmacotherapy in isolation has shown efficacy for the treatment of depression, this study focused on an e-CBT intervention with augmentation options (i.e., e-CBT vs. e-CBT + Call, vs. e-CBT + Call + Pharmacotherapy). Hence, no treatment arm with just pharmacotherapy was included in the treatment intensities.

### 3.5 e-CBT platform

The web-based platform used for the study, the OPTT, is a secure, cloud-based, digital mental health platform (44). It complies with the Health Insurance Portability and Accountability Act, Personal Information Protection and Electronic Documents Act, and Service Organisation Control-2. In addition, all servers and databases are hosted in the Amazon Web Service Canada cloud infrastructure, which is managed by Medstack to ensure that all provincial and federal privacy and security regulations are met. OPTT does not collect any identifiable personal information or internet protocol addresses for privacy purposes. OPTT only collects anonymized metadata to improve its service quality and provide advanced analytics to the clinician team. OPTT encrypts all data, and no employees have direct access to participants’ data. All encrypted backups are kept in the S3 storage that is dedicated to Queen’s University, located in Kingston, Ontario, Canada.

### 3.6 e-CBT program

The e-CBT sessions used in this study include content based on cognitive restructuring and behavioural activation techniques (45). The purpose of the sessions is to help participants become aware of inaccurate or negative thinking patterns so that they can view challenging situations more clearly and respond to them effectively. The sessions prompt participants to understand their situation/environment and the resulting thoughts, behaviours, physical reactions, and feelings. This program aims to help change participants’ negative and/or ineffective thoughts to more effective ways of thinking. As expressed in CBT, changing thoughts can subsequently affect feelings, behaviours, and physical reactions to stressful situations. The 13 e-CBT sessions are outlined below (Table 1).

**Table 1:** Session titles and descriptions for the e-CBT program.

Session	Description
*1 – What is Depression?*	Provides expectations for the course and introduces CBT and depression.
*2 – The 5-Part Model*	Introduces the concept of the 5-Part Model and how a situation, thoughts, feelings, physical reactions, and behaviours are connected and how they interact.
*3 – Sleep Hygiene*	Focuses on sleep habits and provides a variety of tips and strategies to use to increase sleep hygiene and get better rest.
*4 – Strategies for Stressful Situations*	Provides an overview of helpful strategies that can be used in stressful situations including pleasurable activities and helpful breathing techniques.
*5 – Thoughts, Feelings, Behaviour, Physical Reactions & Environment*	Provides a further detailed exploration of the 5 Part Model and how changes in one area can affect the other 4 parts.
*6 – The Thought Record*	Highlights the first three columns of the Thought Record; a tool used to help understand the connection between feelings, behaviours, and thoughts. The first three columns include the situation, followed by the feelings and automatic thoughts associated with the situation.
*7 – Automatic Thoughts*	This delves into the role of automatic thoughts and how they influence feelings. The focus of this session is to understand how to identify automatic thoughts and specifically identify the most dominant idea, or “hot thought” when presented with a stressful situation. Common thinking errors are also discussed in this session.
*8 – Activity Scheduling*	Provides a break from learning about the Thought Record and instead explains how to use an Activity Record; a tool designed to record and plan weekly activities. This session focuses on how tracking activities can inform mood changes and reinforce the scheduling of pleasurable activities.
*9 – Evidence*	Focuses on the fourth and fifth columns of the Thought Record, which is designed to help gather the information that supports or does not support the identified hot thought.
*10 – Alternative & Balanced Thinking*	Focuses on the final two columns of the Thought Record which reflects on the evidence columns to help find an alternative or balanced view of the situation. The last column invites the viewer to re-rate their feelings based on the completion of the Thought Record.
*11 – Experiments*	Explains the importance of conducting experiments to start believing alternative or balanced thoughts from the Thought Record and begin initiating changes in ineffective thinking patterns.
*12 – Action Plans*	Centred around identifying a problem that needs to be solved and provides a framework on how to create a plan for solving the problem.
*13 – Review*	The final session is a review of the course and summarizes the main CBT concepts and tools that have been taught throughout the program.

### 3.7 Care provider

Each participant will be assigned a care provider who will provide feedback for their weekly sessions before the start of their next session. The assigned care provider will be independent of the multi-professional healthcare team that conducted the intake assessment. All care providers are trained in psychotherapy and have experience delivering electronic psychotherapy. They will be informed of each therapeutic session’s aim and content. They will also continue receiving specialized training through webinars, workshops and exercises with feedback provided by the lead psychiatrist on the research team, a trained and licensed psychotherapist (22, 46). All care providers will be supervised by a trained psychotherapist and the lead psychiatrist, and all feedback will be reviewed before submission to the participants.

### 3.8 e-CBT weekly feedback

Weekly homework is reviewed by the independent care provider assigned to the participant, who will provide text-based personalized feedback on OPTT before the next weekly session. Participants and care providers can also communicate asynchronously on OPTT to relay any questions or concerns. The care providers will be provided with sample feedback templates and scripts for the telephone and video call sessions. Templates and scripts will be adapted from previous studies conducted by the research team. Feedback templates and scripts will vary between sessions, and care providers will personalize them for each patient. The feedback templates follow a generic structure starting with, acknowledging the participant’s time and effort since the last session, summarising the CBT concepts taught in the previous session, reviewing the event they explained in their homework, validating the participant’s experience(s), and encouraging the participant to keep up with the sessions. The feedback is written in a letter format to increase personalization and build rapport with the participants.

### 3.9 Questionnaires & follow-up

Participants will complete a series of validated questionnaires at baseline including the Patient Health Questionnaire – 9 Item (PHQ-9), Sheehan Disability Scale (SDS), Quick Inventory of Depressive Symptoms (QIDS), Assessment of Quality of Life (AQoL-8D), and a demographic questionnaire. The PHQ-9 and QIDS questionnaire will be used to assess depressive symptoms and the AQoL-8D questionnaire will assess the participants’ quality of life. These three questionnaires provide insight into the participant’s mental status and perceived quality of life in the context of their depression. Additionally, the SDS will be used to assess the patient’s functional impairment for the clinician group assessment during baseline. During the program, participants will complete the PHQ-9 every 3 weeks (i.e., weeks 4, 7, 10, and 13), and the QIDS and AQoL-8D at weeks 7 and 13. All questionnaires (i.e., PHQ-9, QIDS, and AQoL-8D questionnaires) will be filled at the 3, 6, and 12-month follow-up periods to measure post-study outcomes.

### 3.10 Sample size determination

In our previous clinical trials and gathered data (47), the average PHQ-9 score changed from 16.2 before e-CBT to 11.48 after 12 sessions of e-CBT (joint standard deviation; SD = 5.45). Based on these numbers, the effect size (Hedges’ G) is equal to 0.86. Given the effect size and a power of 0.8, we would need 14 participants to observe a significant effect in a paired sample *t*-test. Considering the completion rate of 45%, we would require 31 participants in each group to observe significant results. Given the two treatment arms and three treatment intensities, we would require 186 participants to detect significant clinical change across all groups. We believe this sample size is large enough to perform further analyses on factors such as the role of sex and gender in treatment efficacy and completion rate.

To follow up on this sample size determination, we will use previously collected data as mentioned above to simulate the population and use a random sampling method to determine the effect size for our power calculation. We will do this by simulating a population based on the mean and standard deviation of our previous e-CBT studies and collecting data, then randomly sampling participants to each treatment arm. This simulation will be developed using Python code. Based on this random sampling, we will calculate the effect size, set the power of 0.8, and consider the completion rate of 45% to determine the sample size required to detect a significant clinical change across the groups.

## 4 Statistical analysis

Initially, all data will be examined for missing, nonsensical, and outlying variables. Missing data will not be attributed and will be treated as missing. All statistical analyses will be performed at the end of the trial and will consider a significance level of 0.05 and 95% confidence intervals. Analyses will be conducted to account for and assess differences across sex and gender identifications, and how these factors affect the adaptability and efficacy of e-CBT.

### 4.1 Hypothesis 1: outcomes in depressive symptoms of treatment arm 1 will be comparable to treatment arms 2 and 3

Clinical outcomes of the healthcare teams’ decision (arm 1) versus the AI’s decision (arm 2), will be compared by assessing the treatment completion rate, number of completed sessions, and change in questionnaire scores. The completion rate across the two study arms and three levels of care will be calculated using a 2 × 3 × 2 contingency matrix (i.e., healthcare team vs. AI × 3 different treatment intensities × completers vs. dropouts) and chi-square analysis to compare within and between completion rates in the two arms. We will use a 2 × 3 (i.e., healthcare team vs. AI × 3 different levels of care) two-way analysis of variance (ANOVA) to compare the number of completed sessions across participant groups and care levels. Questionnaires are collected at 3 different times; at the start (for all participants), midway (for those who completed at least 6 sessions), and end of the study (for those who completed the treatment program). We will use a 2 × 3 × 3 (i.e., healthcare team vs. AI × 3 different levels of care × 3 time points) linear mixed-effects model for each questionnaire to compare clinical outcomes within and between groups and times. A mixed-effects model analysis will account for any missing data caused by participant dropout. Finally, we will assess if demographic covariables affect clinical outcomes using multiple 2 × 2 × 3 × 3 (demographic factors (e.g., male vs. female) × healthcare team vs. AI × 3 different levels of care × 3-time points for the questionnaire’s score) mixed-effects model to account for each demographic variable (i.e., sex, gender, age, and income). Bonferroni corrections will follow the ANOVA analyses in *post hoc* analyses.

### 4.2 Hypothesis 2: AI-based clinical decision-making will provide suggestions comparable to a multi-professional care team

To compare decision-making between the healthcare team (Arm 1) and AI (Arm 2), we will calculate the percentage of identical choices (e.g., arm 1 e-CBT intensity 1 and arm 2 e-CBT intensity) and use a 3 × 3 crosstab analysis (i.e., 3 e-CBT intensities for treatment arm 1 versus 3 e-CBT intensities for treatment arm 2). A chi-square test will be used to evaluate the significance and whether the two models (i.e., healthcare team vs. AI decision) are related or independent. From a machine learning perspective, we will calculate the model’s precision, recall, and F1 score, assuming the healthcare team’s performance level is the baseline level. The precision, recall, and F1 scores provide insight into the quality of the machine-learning model (48).

### 4.3 Hypothesis 3: the AI approach will decrease the time and cost commitment of providing e-CBT

The cost of initial assessments (i.e., manual participant stratification by the healthcare team vs. the AI) and the different care intensities (i.e., only e-CBT, e-CBT + calls, or e-CBT + pharmacotherapy) will also be evaluated. These costs will be evaluated from a health sector and societal perspective (38). For this analysis we will use (1) healthcare costs paid by the government of Ontario as well as healthcare utilization costs self-reported by participants using a resource-use questionnaire (39, 40); (2) medical and productivity costs due to work absence, evaluated using the Questionnaire on Healthcare Consumption and Productivity Losses for patients with a Psychiatric Disorder (TiC-P) (49); (3) intervention costs including staff time-commitment to deliver interventions, development and maintenance costs, and time commitment to assess and enroll participants into the online program. The costs will be expressed in Canadian dollars (CAD). We will use a 2 * 3 (i.e., healthcare team vs. AI * 3 different levels of care) two-way ANOVA to compare costs within and between arms.

## 5 Discussion

Artificial intelligence and providing patients with varying intensities of care can increase the efficiency of mental health care services. This study aims to determine a cost-effective method to decrease depressive symptoms and increase treatment adherence to online psychotherapy by allocating the correct intensity of therapist care for individuals diagnosed with depression. This will be done by comparing a decision-making machine learning algorithm to a multi-professional care team. This approach aims to accurately allocate care tailored to each patient’s needs, allowing for more efficient use of resources with the convergence of technologies and healthcare.

## Ethics statement

The studies involving humans were approved by Queen’s University Health Science and Affiliated Teaching Hospitals Research Ethics Board. The studies were conducted in accordance with the local legislation and institutional requirements. The participants provided their written informed consent to participate in this study.

## Author contributions

All authors listed have made a substantial, direct, and intellectual contribution to the work and approved it for publication.

## Glossary

**Table:** 

AI	Artificial intelligence
ANOVA	Analysis of variance
AQoL-8D	Assessment of Quality of Life – 8 Dimension
CAD	Canadian dollars
CBT	Cognitive behavioural therapy
DSM-5	Diagnostic and Statistical Manual of Mental Disorders – 5th Edition
e-CBT	Electronically delivered cognitive behavioural therapy
MDD	Major depressive disorder
MINI	Mini-International Neuropsychiatric Interview
NLP	Natural language processing
OPTT	Online Psychotherapy Tool
PHQ-9	Patient Health Questionnaire – 9 Item
QALY	Quality-adjusted life years
QIDS	Quick Inventory of Depressive Symptoms
SD	Standard deviation
SDS	Sheehan Disability Scale
TiC-P	Questionnaire on Healthcare Consumption and Productivity Loss for Psychiatric Patients
